# Supplementary material for: Experimental evolution of post-ingestive nutritional compensation in response to a nutrient-poor diet
Source: Proc Biol Sci. 2020 Dec 2;287(1940):20202684. doi: 10.1098/rspb.2020.2684 (PMC7739944; doi:10.1098/rspb.2020.2684)
Supplement: Supplementary Methods, Figures, Tables and R Code from Experimental evolution of post-ingestive nutritional compensation in response to a nutrient-poor diet [file rspb20202684supp1.pdf]

Experimental evolution of post-ingestive nutritional compensation in response to a nutrient-poor diet

Fanny Cavigliasso[1], Cindy Dupuis[1], Loriane Savary[1], Jorge E. Spangenberg[2], Tadeusz J. Kawecki[1,3]

[1] *Department of Ecology and Evolution, University of Lausanne, Lausanne, Switzerland*
[2] *Institute of Earth Surface Dynamics, University of Lausanne, Lausanne, Switzerland*
[3] *Correspondence: tadeusz.kawecki@unil.ch*

Proceedings of the Royal Society B; doi:10.1098/rspb.2020.2684

Electronic Supplementary Material

Supplementary Methods	p. 2
Supplementary Figures S1-S3	p. 6
Supplementary Tables S1-S5	p. 9
R Code	p. 13

Supplementary Methods

Food media in the nutrient assimilation experiment

The main sources of nutrients in the food medium used in the experimental evolution were yeast, cornmeal and sugars (see Methods). However, in the nutrient assimilation experiment we used a medium without cornmeal, with yeast and sucrose as the only sources of nutrients. We did so because cornmeal grains, being relatively coarse, create a degree of heterogeneity in the medium that might allow larvae to prefer or avoid them selectively, and our interest was in post-ingestive rather than pre-ingestive compensation. The absence of cornmeal also made it easier to remove the larvae from the medium. While often used, cornmeal is not a necessary ingredient of *Drosophila* diet.

We used media with different isotope ratio of carbon ($^{13}C/^{12}C$) and nitrogen ($^{15}N/^{14}N$) to evaluate the assimilation of sugar and yeast, respectively. Three different media were used: low $\delta^{15}N$|low $\delta^{13}C$ "standard" medium, low $\delta^{15}N$|low $\delta^{13}C$ "poor" medium and high $\delta^{15}N$|high $\delta^{13}C$ "poor" medium. We first prepared unlabeled and labeled cultured yeast as follows. We grew baker's yeast (starting from a dry form, OD_{600}=100) overnight at 30°C in either unlabeled or labeled SD media (8.5 g yeast nitrogen base w/o AA and w/o ammonium sulfate, 5 g unlabeled ammonium sulfate (or 2.5 g unlabeled ammonium sulfate + 2.5 g labeled ammonium sulfate), 20 g sucrose per liter of water). Unlabeled and labeled cultured yeast were collected by removing the supernatant after centrifugation (5 min at 3000×g) and stored at -20°C. To prepare low $\delta^{15}N$|low $\delta^{13}C$ standard medium, 2.5 mL of unlabeled cultured yeast was added to a solution of 12.5 g of dry yeast dissolved in 100 mL of water before to be added in one liter of water containing 10 g agar, 90 g beet sucrose, 0.5 g $CaCl_2$, 0.5 g $MgSO_4$, 10 mL Nipagin 10%, 6 mL propionic acid, 20 mL ethanol. Low $\delta^{15}N$|low $\delta^{13}C$ poor medium contained one fourth of the amount of sugar and yeast of standard medium. High $\delta^{15}N$|high $\delta^{13}C$ poor medium contained the same amount of sucrose and yeast than low $\delta^{15}N$|low $\delta^{13}C$ poor medium but beet sucrose and unlabeled cultured yeast were replaced by cane sucrose and labeled cultured yeast. After solidification, diets were blended and 10 mL of food were put in petri dishes of 46 mm? diameter.

Protocol of the nutrient assimilation experiment

We transferred approximately 300 eggs to a petri dish with low $\delta^{15}N$|low $\delta^{13}C$ medium and content corresponding to the standard diet. The egg transfer procedure interrupts natural transmission of microbiota, and microbiota can impact host physiology. Therefore, to ensure homogeneity in larva microbiota we collected feces from a pool of flies from all Control and Selected populations and added onto the medium (100 µL of phosphate-buffered saline (PBS) suspension at OD_{600} = 0.5)[31]. Sixty hours later we collected two groups of 80 to 90 larvae from each replicate. The first group was transferred to a petri dish containing low $\delta^{15}N$|low $\delta^{13}C$ poor medium, the second group to one containing high $\delta^{15}N$|high $\delta^{13}C$ poor medium. Twenty-four hours after the transfer, we transferred the larvae for 3h to petri dishes containing agar (1%) covered by filter paper to allow the larvae to empty their guts; this was done to ensure that isotopic measurement reflects what was assimilated in tissues rather than gut content. (The 3h time to empty gut followed [38] and was verified in our flies, see below.) Approximately 50 larvae from each group were then flash-frozen and stored at -80°C until the isotope measurement. The experiment was carried out in three blocks, each with one replicate per population.

Estimation of time needed to empty larval gut

Because isotopic measurement was done on whole larva and to ensure that what we measured reflects what was absorbed in tissues and not what remained in gut, we needed to starve larvae to empty their gut before their collection. Therefore we estimated the food retention time in gut larvae. For this, we fed 2.5 days-old larvae on blue-stained media (either low $\delta^{15}N$|low $\delta^{13}C$ or high $\delta^{15}N$|high $\delta^{13}C$ poor medium; 100 mL of poor medium were stained with 1.25 mL of Erioglaucine 0.2%) during 24h. Then, we transferred 25 larvae per population and medium to small petri dish containing agar (1%) covered by filter paper in order to starve larvae and measure the time necessary to empty their gut. Each hour, and half hour from 2h to 3h30, the number of dead larvae, uncolored larvae (=empty larvae) and colored larvae were counted. At each measure, we removed dead larvae from the experiment.

We did not observe blue dye after 2h of starvation for 25% of larvae (Figure S1A and S1B). This proportion increased over time with a maximal slope observed after 2h30 and 3h of starvation. After 3h, most of the gut of larvae were empty or with less than 25% of blue dye remaining in the gut (Supplementary Figure S1A and S1B).

We then tested if the proportion of larvae without blue dye remaining in their gut after 3h of starvation was the same between Control and Selected populations and between diets. For this we fitted a generalized linear mixed model (GLMM) with a binomial error distribution was fitted with the number of larvae with empty gut versus the number of larvae with blue dye in gut as the response variable, the selection regime, the diet and the interaction of both as fixed factors, and replicate populations and its interaction with diets as random factors.

Although a very little blue dye remained in some larvae, the proportion did not differ between Control and Selected populations (Figure S1C, $\chi^2 = 0.5$, $p = 0.50$) nor between diets ($\chi^2 = 0.0$, $p = 0.94$). Based on this assay we decided to starve all larvae during 3h to remove most of the nutrients from the gut before the measurement of isotope ratio.

Measurement of the isotope ratios

The carbon and nitrogen stable isotope compositions were determined by elemental analysis/isotope ratio mass spectrometry (EA/IRMS), using a Carlo Erba 1108 (Fisons Instruments, Milan, Italy) elemental analyzer connected to a Delta V Plus isotope ratio mass spectrometer via a ConFlo III split interface (both of Thermo Fisher Scientific, Bremen, Germany) operated under continuous helium (He) flow[39,40]. The stable isotope compositions were reported in the delta (*d*) notation as per mil (‰) variations of the molar ratio (*R*) of the heavy (^{h}E) to light isotope (^{l}E) of element E (i.e., $^{13}C/^{12}C$ and $^{15}N/^{14}N$) relative to an international standard:

$$\delta^{i}E_{sample/standard} = \frac{R(\,^{h}\mathrm{E}/\,^{l}\mathrm{E})_{sample}}{R(\,^{h}\mathrm{E}/\,^{l}\mathrm{E})_{standard}} - 1$$

For carbon stable isotope ratios ($\delta\,^{13}C$), the standard is the Vienna Pee Dee Belemnite limestone (VPDB); for nitrogen ($\delta^{15}N$), the standard is molecular nitrogen in air (Air-N_2). For

calibration and normalization of the measured isotopic ratios to the international scales (VPDB-LSPVEC lithium carbonate scale for $\delta^{13}C$, Air-N_2 scale for $\delta^{15}N$), a 3- or 4-point calibration was used with international reference materials (RMs) and UNIL in-house standards [40]. The repeatability and intermediate precision of the EA/IRMS analyses of must sugars and wine solid residues were determined by the standard deviation of separately replicated analyses and were better than 0.05 and 0.1 mUr for $\delta^{13}C$ and $\delta\ ^{15}N$, respectively. The carbon and nitrogen concentrations (in wt.%) were determined from the peak areas of the major isotopes using the calibrations for $\delta^{13}C_{VPDB}$ and $\delta^{15}N_{Air\text{-}N2}$. The repeatability was better than 0.2 wt.% for carbon and nitrogen contents.

Quantification of triglyceride content in the prepupae

Quantification of TAG was based on the measurement of the amount of free glycerol released after TAG digestion by lipase. The samples were homogenized in 250 µL of TE buffer (10 mM Tris, 1 mM EDTA, pH 8.9) before being heated for 10 min at 70°C. From this step, we followed protocol of [41], except we used TE buffer instead of PBS. Briefly, for TAG assays each homogenate was divided in two and incubated either with TE buffer or TAG reagent containing lipase (Sigma, T2449) at 37°C during 1 h. The incubation with TE buffer allowed us to evaluate the background amount of glycerol, whereas the incubation with enzyme allowed us to evaluate the total glycerol (background + that released after TAG digestion). After incubation, samples were centrifuged (3 min at 13500 rpm) and their absorbance measured at 540 nm. Finally, we subtracted the absorbance values of samples incubated with TE buffer from those incubated with lipase to estimate the TAG content.

After the centrifugation and before the heat treatment, 10 µl of homogenate was used for protein estimation to normalize TAG content. Total protein content was estimated by measurement of the absorbance of the homogenized sample at 280 nm and its conversion to protein content by using a calibration curve obtained from protein standards (BSA, Sigma P5369).

Details of statistical analysis

We performed all statistical analysis with R software (version 3.5.0) and RStudio plugin (version 1.1.453). To test whether carbon and nitrogen assimilation differed between Control and Selected populations, we fitted linear mixed models (LMM, *lmer* function implemented in lmerTest R packages [42]) with nitrogen or carbon assimilation as the response variable, the selection regime and experimental block as fixed factors, and the replicate populations as a random factor. Additionally, to test for differences in basal levels of $\delta^{13}C$ and $\delta^{15}N$ values, we fitted LMM with same set of fixed and random factors to values of $\delta^{13}C$ or $\delta^{15}N$ from larvae transferred to low $\delta^{15}N$|low $\delta^{13}C$ medium.

To test whether fat storage differed between Control and Selected populations, we fitted LMM with the logarithm of the ratio of TAG to protein as the response variable, the selection regime as a fixed factor and the replicate populations nested within selection regime as random factor.

To test whether different amounts of yeast and sugar in diet affected the egg-to-adult survival probability and the adult sex-ratio of survivors, we fitted generalized linear model

(GLMM) with a binomial error distribution, using the *mixed* function implemented in afex R packages [43]. The response variables were the total number of flies *versus* the number of non-emerged flies (number of deposited eggs minus the total number of flies), and the number of females *versus* males. To test whether diets affected sex-specific developmental rate and adult dry weight, we fitted linear mixed models (LMM) with the *lmer* function implemented in lmerTest R package [42], using sex-specific means of developmental rate and weight per replicate rearing bottle as the response variables. In all models the selection regime, supplemental yeast, supplemental sugar, along with their interactions, block and experimenter as fixed effects; and replicate populations, its interactions with supplemental yeast and sugar and the replicate rearing bottle as random factors. *overdisp* function implemented in the sjstats package was used to check for overdispersion. For developmental rate and adult dry weight models, we added sex and its interactions with diets and regime as fixed effects and its interactions with the replicate populations as random factors. From these complete models, we fitted reduced models without interactions that were far from significant (with $p > 0.15$) and with sex separately because at least one interaction involving the sex factor was significant in the developmental rate complete model and dry weight complete model (see Table S3 for results of the complete models). Then, for all models, we tested contrasts of significant interactions involving supplemental sugar or yeast and we compared the condition with supplemental yeast only to the condition with supplemental sugar only with *emmeans* and *pairs* functions in R [44].

Significance of LMM fixed factors in all models were tested with Type 3 F-tests with Satterthwaite's degrees-of-freedom method. We verified the normality of residuals with Q-Q plots and the Shapiro-Wilk test (*shapiro.test*). Significance of GLMM fixed factors were tested with Likelihood Ratio Test (LRT) method. For cases of non-rejection of the null hypothesis we report 95% confidence intervals for the effect sizes, estimated with the *emmeans* function.

R code for model fitting, significance tests and marginal means estimation is provided at the end of this document

Supplementary Figures

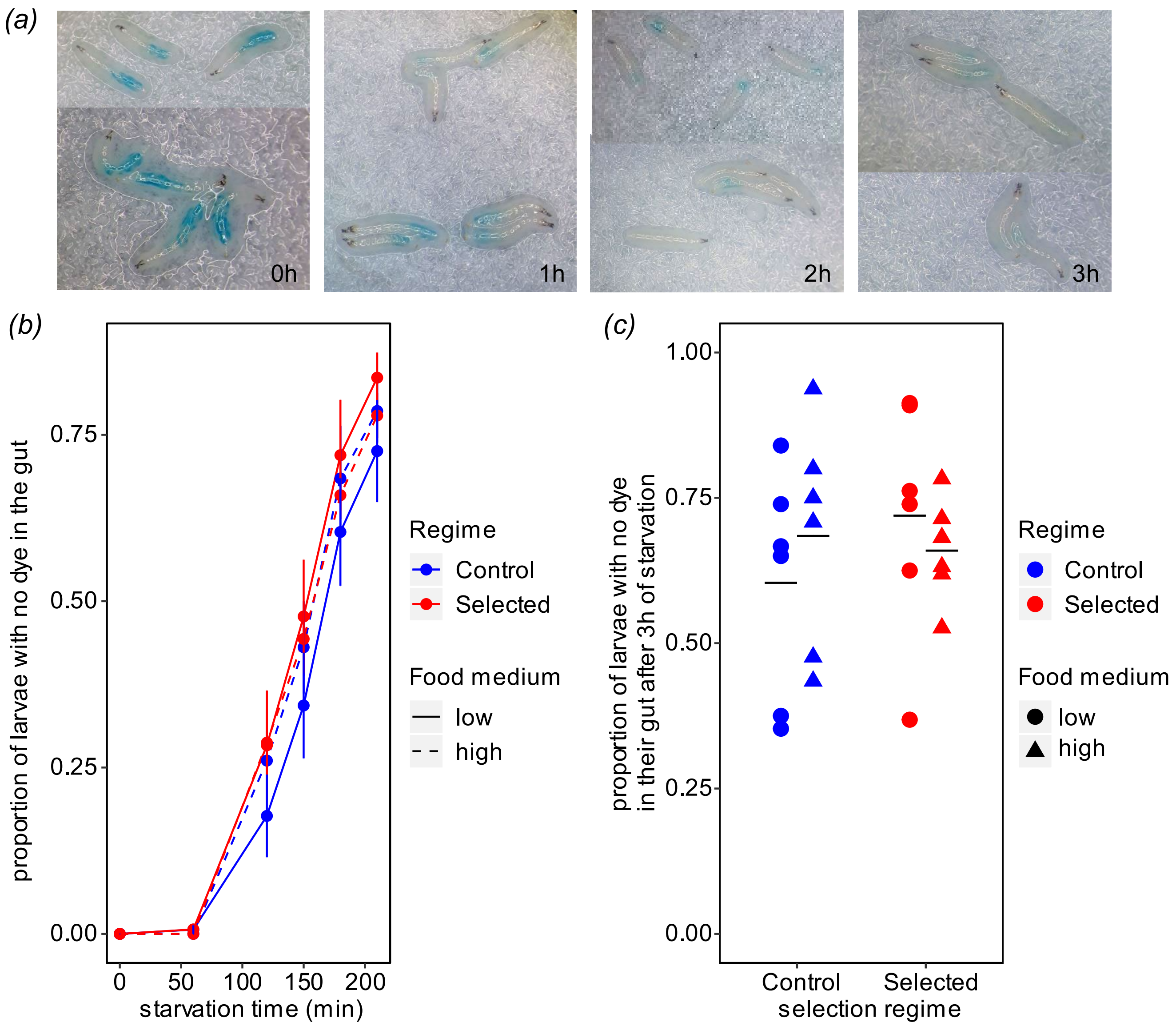

Supplementary Figure S1. Food retention time in the gut of larvae. (a) Images of *Drosophila* larvae previously fed with diet with blue dye after 0, 1h, 2h or 3h of starvation. (b) Proportion of larvae with no remaining blue dye in their gut depending of the starvation duration. Larvae were fed either with blue low $\delta^{15}N$|low $\delta^{13}C$ ("low") or high $\delta^{15}N$|high $\delta^{13}C$ ("high") poor medium. The error bars represent standard errors. (c) Proportion of larvae with no dye in their gut remaining after 3 hours of starvation. Black lines represent the means per selection regime and diet.

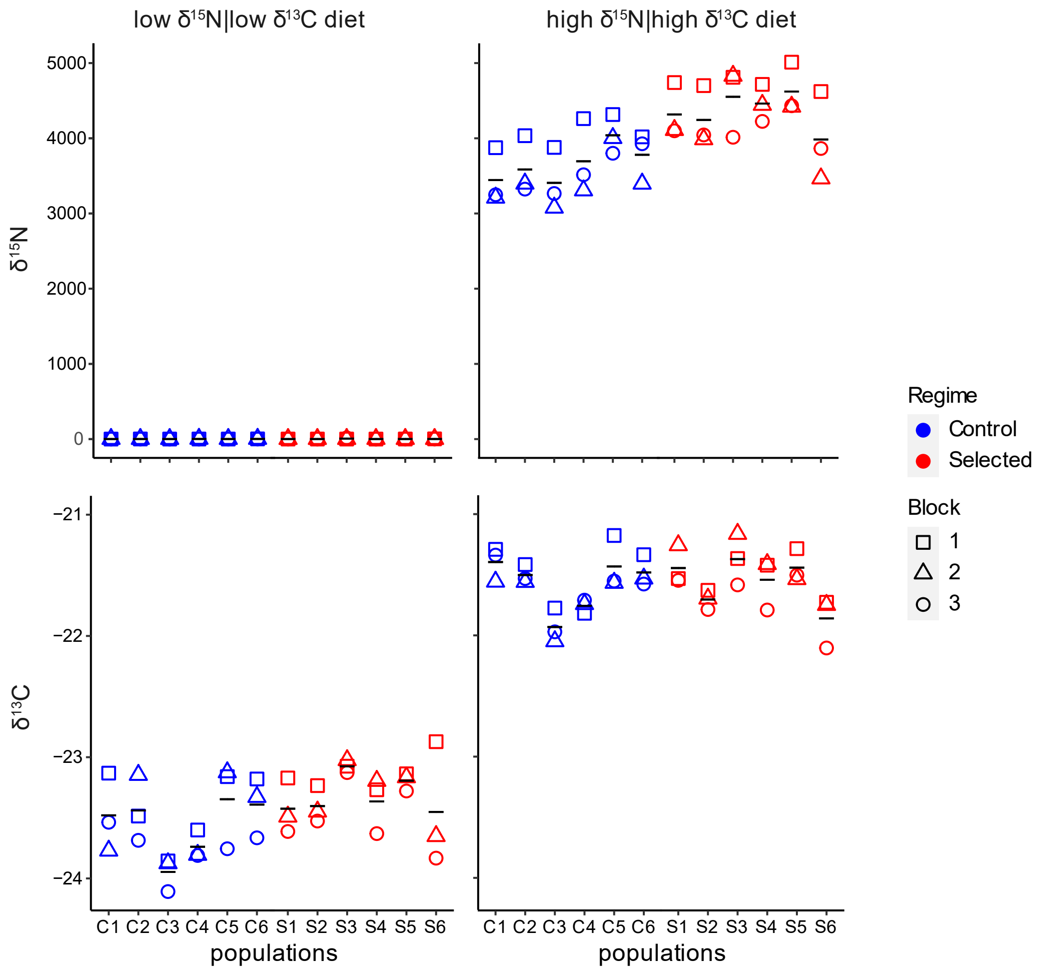

Supplementary Figure S2. Nitrogen (top) and carbon (bottom) isotope ratios measured in the larvae of Control (C1-C6) and Selected (S1-S6) populations in the nutrient assimilation experiment; these numbers are the basis of assimilation rates reported in Figure 1 b,c. Left: larvae transferred to the low $\delta^{15}N$|low $\delta^{13}C$ medium (i.e., basal level of $\delta^{15}N$ or $\delta^{13}C$); right: larvae transferred to high $\delta^{15}N$|high $\delta^{13}C$ medium. Each colored point corresponds to one biological replicate (symbol shape reflects experimental block). Black lines indicate the mean for each population.

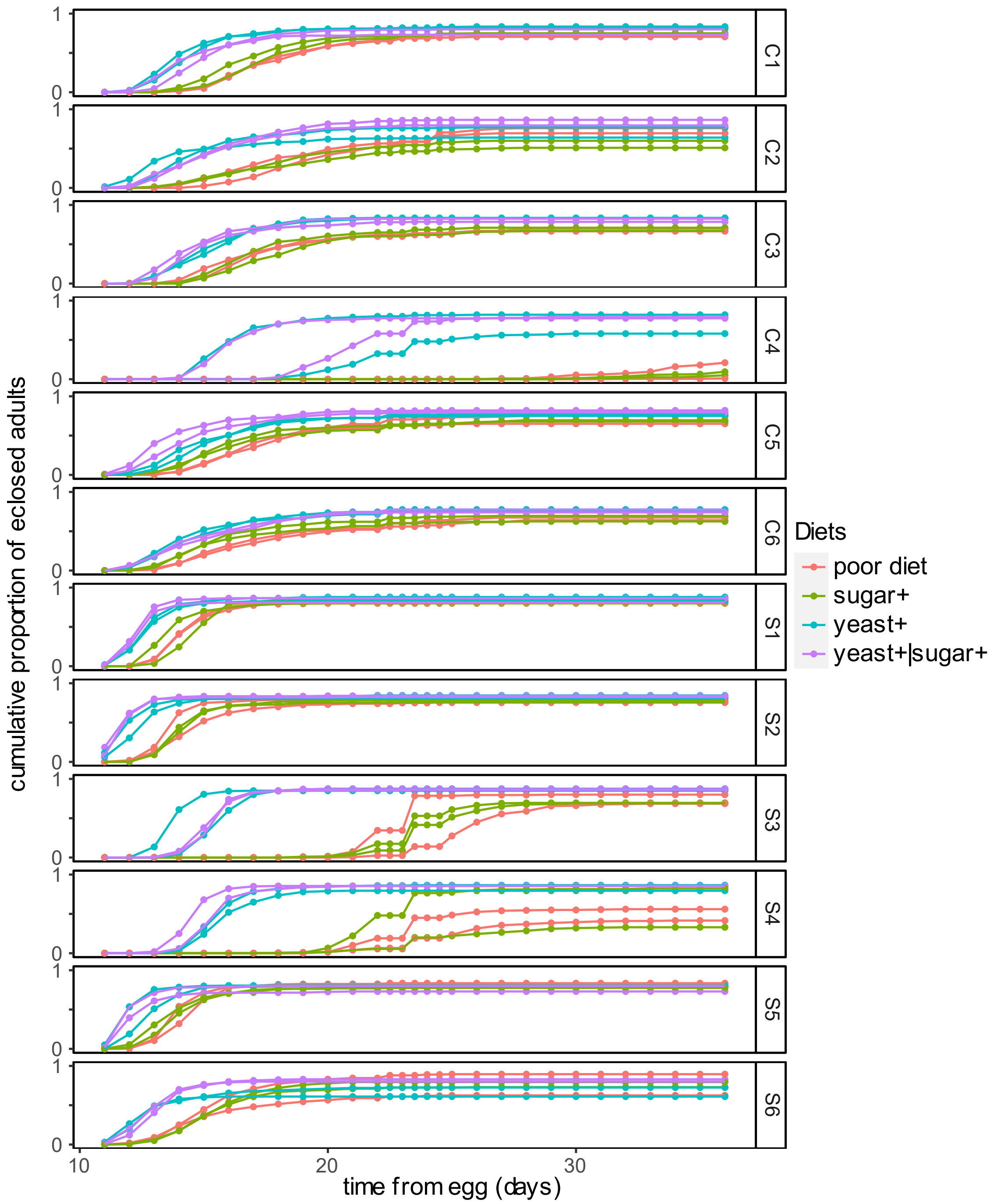

Supplementary Figure S3. Emergence curves of eclosing adult flies in the limiting nutrient experiment. C1 to C6: Control populations; S1 to S6: Selected populations. Each curve corresponds to the cumulative proportion of emerging flies per bottle. Colored indicate the diet (two lines of the same color correspond to two replicate culture bottles). C1, C2, S1 and S2 populations formed the first experimental block; C3, C4, S3 and S4 the second, and C5, C6, S5 and S6 populations the third. Three populations form the second block has abnormally delayed development; this block was thus excluded from the analysis (see Methods).

Supplementary Tables

Supplementary Table S1: Average values of Total Organic Carbon (TOC), $\delta^{13}C$ and $\delta^{15}N$ measured in media, sucrose and yeast. SD: standard deviation. NA: not measured. Yeast values = 0.1 x (mean(Unlabeled or Labeled lab grown yeast)) + 0.9 x (mean(commercial yeast)).

Sample type	TOC		$\delta^{13}C$		$\delta^{15}N$	
	Mean (%)	SD	Mean (‰)	SD	Mean (‰)	SD
Low $\delta^{15}N$|low $\delta^{13}C$ standard medium	41.69	0.50	-25.51	0.11	1.15	0.60
Low $\delta^{15}N$|low $\delta^{13}C$ poor medium	41.82	0.65	-24.73	0.14	4.94	3.26
High $\delta^{15}N$|high $\delta^{13}C$ poor medium	41.45	0.41	-16.47	0.09	6691.51	266.13
Cane sugar	42.03	0.71	-12.69	0.06	NA	NA
Beet sugar	42.33	0.35	-26.47	0.04	NA	NA
Unlabeled yeast	42.32	3.00	-23.22	0.06	NA	NA
Labeled yeast	42.23	3.01	-23.21	0.06	NA	NA

Supplementary Table S2: Type 3 likelihood ratio tests for fixed effects in generalized linear mixed model on proportion of larvae with no remaining blue dye in their gut after 3h of starvation, reported in Figure S1c.

Factor	Df	χ^2	*df*	*P*
Regime	5	0.45	1	0.50
Diet	5	0.00	1	0.94
Regime × Diet	5	1.38	1	0.24

Supplementary Table S3: Type 3 likelihood ratio tests for fixed effects in complete generalized linear mixed models on egg-to-adult survival probability and adult sex ratio. Type 3 F-tests with Satterthwaite's method for fixed effects in linear mixed effect models on developmental rate and adult dry weight. Supp.: Supplemental. Significant P-values (*P* < 0.05) are in bold.

	Egg-to-adult survival			Adult Sex ratio			Developmental rate			Adult dry weight		
Factor	*df*	χ^2	*P*	*df*	χ^2	*P*	*df*	*F*	*P*	*df*	*F*	*P*
Regime	1,14	13.0	**<0.001**	1,14	0.3	0.56	1,4	50.47	**0.002**	1,4	152.82	**<0.001**
Supplemental yeast	1,14	13.4	**<0.001**	1,14	0.6	0.45	1,6	154.60	**<0.001**	1,37.7	229.79	**<0.001**
Supplemental sugar	1,14	3.0	0.083	1,14	1.5	0.22	1,6	1.66	0.25	1,25.7	0.03	0.87
Sex							1,6	6.28	**0.046**	1,6	258.98	**<0.001**
Block	1,14	3.7	0.055	1,14	0.4	0.53	1,4	0.00	0.97	1,4	0.24	0.65
Experimenter	1,14	6.2	**0.012**	1,14	2.1	0.15	1,4	0.46	0.54	1,4	0.19	0.68
Regime × Supp. yeast	1,14	8.8	**0.003**	1,14	2.3	0.13	1,6	0.59	0.47	1,37.7	0.40	0.53
Regime × Supp. sugar	1,14	0.1	0.75	1,14	1.9	0.17	1,6	3.44	0.11	1,25.7	0.02	0.89
Supp. yeast × Supp. sugar	1,14	0.0	0.98	1,14	0.5	0.49	1,6	3.31	0.12	1,37.7	13.78	**<0.001**
Regime × Sex							1,6	0.81	0.40	1,6	8.21	**0.029**
Supp. yeast × Sex							1,44	6.58	**0.014**	1,17	6.22	**0.023**
Supp. sugar × Sex							1,6	0.01	0.94	1,7.9	0.70	0.43
Regime × Supp. yeast × Supp. sugar	1,14	0.1	0.74	1,14	0.0	1.00	1,6	0.16	0.70	1,37.7	1.42	0.24
Regime × Supp. yeast × Sex							1,44	0.02	0.887	1,17	0.28	0.61
Regime × Supp. sugar × Sex							1,6	2.39	0.17	1,7.9	2.14	0.18
Supp. yeast × Supp. sugar × Sex							1,44	0.00	0.98	1,17	7.33	**0.015**
Regime × Supp. yeast × Supp. sugar × Sex							1,44	0.46	0.50	1,17	0.06	0.81

Supplementary Table S4: Summary of type 3 likelihood ratio tests and F-tests for fixed effects in linear mixed models on four traits reported in Figure 2. Contrasts of significant interactions involving supplemental sugar or yeast are reported. Supp.: Supplemental. – yeast or – sugar: No supplemental yeast or sugar. + yeast or + sugar: Supplemental yeast or sugar. Significant P-values ($P < 0.05$) are in bold.

	Egg-to-adult survival		Adult sex ratio		Developmental rate				Dry weight			
					Female		Male		Female		Male	
Factors	Statistics	*P*	Statistics	*P*	Statistics	*P*	Statistics	*P*	Statistics	*P*	Statistics	*P*
Regime	$\chi^2_{1,13} = 13.0$	**<0.001**	$\chi^2_{1,11} = 0.4$	0.54	$F_{1,4} = 43.4$	**0.003**	$F_{1,4} = 40.1$	**0.003**	$F_{1,4} = 73.8$	**0.001**	$F_{1,11} = 356.2$	**<0.001**
Supp. yeast	$\chi^2_{1,13} = 13.8$	**<0.001**	$\chi^2_{1,11} = 0.6$	0.46	$F_{1,7} = 140.4$	**<0.001**	$F_{1,7} = 194.1$	**<0.001**	$F_{1,21} = 184.4$	**<0.001**	$F_{1,46} = 220.9$	**<0.001**
Supp. sugar	$\chi^2_{1,13} = 3.0$	0.082	$\chi^2_{1,11} = 1.7$	0.19	$F_{1,6} = 1.2$	0.31	$F_{1,6} = 1.7$	0.24	$F_{1,21} = 0.1$	0.74	$F_{1,11} = 0.5$	0.48
Block	$\chi^2_{1,13} = 3.6$	0.056	$\chi^2_{1,11} = 0.4$	0.51	$F_{1,4} = 0.01$	0.91	$F_{1,4} = 0.0$	0.96	$F_{1,4} = 0.01$	0.95	$F_{1,11} = 2.3$	0.16
Experimenter	$\chi^2_{1,13} = 6.2$	**0.013**	$\chi^2_{1,11} = 2.1$	0.15	$F_{1,4} = 0.7$	0.46	$F_{1,4} = 0.2$	0.71	$F_{1,4} = 0.06$	0.81	$F_{1,11} = 0.7$	0.43
Regime x Supp. yeast	$\chi^2_{1,13} = 9.0$	**0.003**	$\chi^2_{1,11} = 2.4$	0.12								
Regime x Supp. sugar					$F_{1,6} = 4.33$	0.083	$F_{1,6} = 1.63$	0.25				
Supp. yeast x Supp. sugar					$F_{1,7} = 3.11$	0.12	$F_{1,7} = 4.16$	0.081	$F_{1,21} = 19.76$	**<0.001**	$F_{1,46} = 4.48$	**0.040**
Pairwise contrasts												
– yeast vs + yeast												
in Control populations	$z = -5.2$	**<0.001**										
in Selected populations	$z = -0.6$	0.532										
– yeast vs + yeast												
in No supp. sugar									$t_{14} = -12.7$	**<0.001**	$t_{14} = -12.0$	**<0.001**
in Supp. sugar									$t_{14} = -6.5$	**<0.001**	$t_{14} = -9.0$	**<0.001**
– sugar vs + sugar												
in No supp. yeast									$t_{10.6} = -2.9$	**0.012**	$t_{13.8} = -1.2$	0.071
in Supp. yeast									$t_{10.6} = 3.4$	**0.005**	$t_{13.8} = 0.9$	0.40

Supplementary Table S5: Pairwise comparisons between effects of supplemental yeast only ('yeast+') and supplemental sugar only ('sugar+') on performance traits reported in Figure 2. Difference: Improved performance when yeast is added alone, compared to sugar supplement alone. For egg-to-adult survival probability, results were averaged over the levels of experimenter and day. For developmental rate and adult dry weight, results were averaged over the levels of regime, experimenter and day. *P*-values were adjusted with the Tukey approach.

Contrast	Variable	Regime or Sex	Difference (%)	Estimate	SE	statistics	*P*
yeast+ vs sugar+	Egg-to-adult survival probability	Control	19.6	0.636	0.123	z = 5.2	<0.001
		Selected	3.7	0.205	0.126	z = 1.6	0.36
	Developmental rate	Female	15.5	9.66e-03	1.16e-03	$t_{12.9} = 8.3$	<0.001
		Male	13.6	8.73e-03	9.09e-04	$t_{13} = 9.6$	<0.001
	Adult Dry Weight	Female	25.1	0.040	4.09e-03	$t_{14} = 9.8$	<0.001
		Male	24.1	0.030	3.19e-03	$t_{13.8} = 9.4$	<0.001

R code for statistical analysis

Nitrogen and carbon assimilation

```
read.table("assimilation_data.txt",h=T)->abs_all
droplevels(subset(abs_all,condition=="L_High"))->droso_sample
# Nitrogen: assimilation
lmer(Nitrogen_assimilation~regime +block+(1|pop_name), data = droso_sample, REML = F)->lm_N1
anova(lm_N1)
#Nitrogen: basal level
lmer(d15N~regime+block+(1|pop_name), data = abs_all[abs_all$condition=="L_Low",],REML = F)-
>lm_d15N_larvae
anova(lm_d15N_larvae)
# Carbon: assimilation
lmer(Carbon_absorption~regime +block+(1|pop_name), data = droso_sample, REML = F)->lm_C1
anova(lm_C1)
# Carbon: basal level
lmer(d13C~regime+block+(1|pop_name), data = abs_all[abs_all$condition=="L_Low",],REML = F)-
>lm_d13C_larvae
anova(lm_d13C_larvae)
```

Accumulation of triglycerides

```
read.table("TAG prepupae.txt",h=T)->TAG
lmer(logtagprot~Regime+(1|Regime/Population), data = TAG, REML = F)->lm_TAG_poor
qqnorm(resid(lm_TAG_poor))
qqline(resid(lm_TAG_poor))
shapiro.test(resid(lm_TAG_poor))
anova(lm_TAG_poor)
```

Limiting nutrient for larval fitness traits

```
read.table("limiting_nutrient.txt",h=T)->KPF_KMF
droplevels(subset(KPF_KMF,sex=="Total"))->KPF_KMF_clean_total
droplevels(subset(KPF_KMF,!sex=="Total"))->KPF_KMF_clean_sex
```

<u># Survival rate</u>

```
head(KPF_KMF_clean_total)
200-KPF_KMF_clean_total$total->KPF_KMF_clean_total$dead
glmer(cbind(total,dead)~regime*Add_yeast*Add_sugar + experimenter  + block  + (1|
pop_name)+(1|Add_yeast: pop_name)+(1|Add_sugar: pop_name)+(1|Add_yeast:Add_sugar:
pop_name)+(1| number), data =KPF_KMF_clean_total,family = "binomial") ->glm_surv_tot5
overdisp(glm_surv_tot5)
# dispersion ratio = 0.4590
# Pearson's Chi-Squared = 22.4889
```

```
# p-value = 0.9996
mixed(cbind(total,dead)~regime*Add_yeast*Add_sugar+ experimenter +block+  (1|
pop_name)+(1|Add_yeast: pop_name)+(1|Add_sugar: pop_name)+(1|Add_yeast:Add_sugar:
pop_name)+(1| number) , data =KPF_KMF_clean_total,family = "binomial", method="LRT") -
>glm_surv_tot6
anova(glm_surv_tot6)

# reduced model (without interaction with p > 0.15)
glmer(cbind(total,dead)~regime+Add_yeast+Add_sugar+ regime:Add_yeast+experimenter +block+  (1|
pop_name)+(1|Add_yeast: pop_name)+(1|Add_sugar: pop_name)+(1| number) , data
=KPF_KMF_clean_total,family = "binomial",control=glmerControl(optimizer="bobyqa",
optCtrl=list(maxfun=100000))) ->glm_surv_tot8
overdisp(glm_surv_tot8)
# dispersion ratio = 0.4212
# Pearson's Chi-Squared = 22.3215
# p-value = 0.9999
mixed(cbind(total,dead)~regime+Add_yeast+Add_sugar+ regime:Add_yeast+experimenter +block+  (1|
pop_name)+(1|Add_yeast: pop_name)+(1|Add_sugar: pop_name)+(1| number) , data
=KPF_KMF_clean_total,family = "binomial", method="LRT",control=glmerControl(optimizer="bobyqa",
optCtrl=list(maxfun=100000))) ->glm_surv_tot8
anova(glm_surv_tot8)

# Contrasts
emmeans(glm_surv_tot8,c("Add_yeast"),by=c("regime"))->glm_surv_tot9
pairs(glm_surv_tot9)
emmeans(glm_surv_tot8,c("Add_yeast","Add_sugar"),by=c("regime"))->glm_surv_tot9
pairs(glm_surv_tot9)

# Sex ratio
head(KPF_KMF_clean_sex)
droplevels(subset(KPF_KMF_clean_sex,sex=="Fem"))->KPF_KMF_fem
droplevels(subset(KPF_KMF_clean_sex,sex=="Male"))->KPF_KMF_male
cbind(KPF_KMF_fem[,c(1:8,12,21,22)],KPF_KMF_male$total)->KPF_KMF_fem_male
head(KPF_KMF_fem_male)
colnames(KPF_KMF_fem_male)<-c("number","pop","pop_name","regime","Food","bottle","block",
                    "experimenter","total_fem","Add_yeast","Add_sugar","total_male")
head(KPF_KMF_fem_male)

glmer(cbind(total_fem,total_male)~regime*Add_yeast*Add_sugar + experimenter  + block  + (1|
pop_name)+(1|Add_yeast: pop_name)+(1|Add_sugar: pop_name)+(1|Add_yeast:Add_sugar:
pop_name)+(1| number), data =KPF_KMF_fem_male,family = "binomial") ->glm_sex_ratio3
overdisp(glm_sex_ratio3)
# dispersion ratio = 1.1136
# Pearson's Chi-Squared = 54.5653
```

```
# p-value = 0.2713
mixed(cbind(total_fem,total_male)~regime*Add_yeast*Add_sugar+ experimenter +block+  (1|
pop_name)+(1|Add_yeast: pop_name)+(1|Add_sugar: pop_name)+(1|Add_yeast:Add_sugar:
pop_name)+(1| number) , data =KPF_KMF_fem_male,family = "binomial", method="LRT") -
>glm_sex_ratio4
anova(glm_sex_ratio4)

# reduced model (without interaction with p > 0.15)
glmer(cbind(total_fem,total_male)~regime+Add_yeast+Add_sugar+
regime:Add_yeast+block+experimenter +  (1| pop_name)+(1|Add_yeast: pop_name)+(1|Add_sugar:
pop_name)+(1|Add_yeast:Add_sugar: pop_name)+(1| number) , data =KPF_KMF_fem_male,family =
"binomial") ->glm_sex_ratio4
overdisp(glm_sex_ratio4)
# dispersion ratio = 1.0936
# Pearson's Chi-Squared = 56.8698
# p-value = 0.2986
mixed(cbind(total_fem,total_male)~regime+Add_yeast+Add_sugar+
regime:Add_yeast+block+experimenter +  (1| pop_name)+(1|Add_yeast: pop_name)+(1|Add_sugar:
pop_name)+(1|Add_yeast:Add_sugar: pop_name)+(1| number) , data =KPF_KMF_fem_male,family =
"binomial", method="LRT") ->glm_sex_ratio4
anova(glm_sex_ratio4)

# Developmental rate
head(KPF_KMF_clean_sex)
lmer(mean_dev_rate~regime*Add_yeast*Add_sugar*sex+ block + experimenter + (1| pop_name)
+(1|Add_yeast:pop_name)+(1|Add_sugar:pop_name)+(1|Add_yeast:Add_sugar:pop_name)+(1|sex:po
p_name)+ (1|sex:Add_yeast:pop_name)+(1|sex:Add_yeast:Add_sugar:pop_name)
+(1|sex:Add_sugar:pop_name) +  (1|number), data =KPF_KMF_clean_sex)->lm_dev_rate3
plot(lm_dev_rate3)
qqnorm(resid(lm_dev_rate3))
qqline(resid(lm_dev_rate3))
shapiro.test(resid(lm_dev_rate3))
anova(lm_dev_rate3)

# reduced model (without interaction with p > 0.15 and sex separated)
droplevels(subset(KPF_KMF_clean_sex,sex=="Fem"))->KPF_KMF_fem
droplevels(subset(KPF_KMF_clean_sex,sex=="Male"))->KPF_KMF_male

# Females
lmer(mean_dev_rate~regime+Add_yeast+Add_sugar+regime:Add_sugar+Add_yeast:Add_sugar  + block
+ experimenter + (1| pop_name)
+(1|Add_yeast:pop_name)+(1|Add_sugar:pop_name)+(1|Add_yeast:Add_sugar:pop_name), data
=KPF_KMF_fem)->lm_dev_rate_fem
plot(lm_dev_rate_fem)
```

```
qqnorm(resid(lm_dev_rate_fem))
qqline(resid(lm_dev_rate_fem))
shapiro.test(resid(lm_dev_rate_fem))
anova(lm_dev_rate_fem)

emmeans(lm_dev_rate_fem,c("Add_sugar"))->lm_dev_rate_fem_sugar
pairs(lm_dev_rate_fem_sugar)->lm_dev_rate_fem_sugar2
summary(lm_dev_rate_fem_sugar2,infer=c(TRUE,TRUE))

emmeans(lm_dev_rate_fem,c("Add_yeast","Add_sugar"))->lm_dev_rate_fem2
pairs(lm_dev_rate_fem2)

# Males
lmer(mean_dev_rate~regime+Add_yeast+Add_sugar+regime:Add_sugar+Add_yeast:Add_sugar  + block
+ experimenter + (1| pop_name)
+(1|Add_yeast:pop_name)+(1|Add_sugar:pop_name)+(1|Add_yeast:Add_sugar:pop_name), data
=KPF_KMF_male)->lm_dev_rate_male
plot(lm_dev_rate_male)
qqnorm(resid(lm_dev_rate_male))
qqline(resid(lm_dev_rate_male))
shapiro.test(resid(lm_dev_rate_male))
anova(lm_dev_rate_male)

emmeans(lm_dev_rate_male,c("Add_sugar"))->lm_dev_rate_male_sugar
pairs(lm_dev_rate_male_sugar)->lm_dev_rate_male_sugar2
summary(lm_dev_rate_male_sugar2,infer=c(TRUE,TRUE))

emmeans(lm_dev_rate_male,c("Add_yeast","Add_sugar"))->lm_dev_rate_male2
pairs(lm_dev_rate_male2)

# Adult dry weight
head(KPF_KMF_clean_sex)
lmer(weight_per_fly~regime*Add_yeast*Add_sugar*sex+ block + experimenter + (1| pop_name)
+(1|Add_yeast:pop_name)+(1|Add_sugar:pop_name)+(1|Add_yeast:Add_sugar:pop_name)+(1|sex:po
p_name)+ (1|sex:Add_yeast:pop_name)+(1|sex:Add_yeast:Add_sugar:pop_name)
+(1|sex:Add_sugar:pop_name) +  (1|number), data =KPF_KMF_clean_sex)->lm_weight3
plot(lm_weight3)
qqnorm(resid(lm_weight3))
qqline(resid(lm_weight3))
shapiro.test(resid(lm_weight3))
anova(lm_weight3)

# reduced model (without interaction with p > 0.15 and sex separated)
droplevels(subset(KPF_KMF_clean_sex,sex=="Fem"))->KPF_KMF_fem
```

```
droplevels(subset(KPF_KMF_clean_sex,sex=="Male"))->KPF_KMF_male

# Females
lmer(weight_per_fly~regime+Add_yeast+Add_sugar+ Add_yeast:Add_sugar+ block + experimenter + (1|
pop_name)
+(1|Add_yeast:pop_name)+(1|Add_sugar:pop_name)+(1|Add_yeast:Add_sugar:pop_name), data
=KPF_KMF_fem)->lm_weight_fem
plot(lm_weight_fem)
qqnorm(resid(lm_weight_fem))
qqline(resid(lm_weight_fem))
shapiro.test(resid(lm_weight_fem))
anova(lm_weight_fem)

emmeans(lm_weight_fem,c("Add_yeast","Add_sugar"))->lm_weight_fem2
pairs(lm_weight_fem2)

emmeans(lm_weight_fem,c("Add_yeast"),by=c("Add_sugar"))->lm_weight_fem2
pairs(lm_weight_fem2)

emmeans(lm_weight_fem,c("Add_sugar"),by=c("Add_yeast"))->lm_weight_fem2
pairs(lm_weight_fem2)

# Males
lmer(weight_per_fly~regime+Add_yeast+Add_sugar+ Add_yeast:Add_sugar+ block + experimenter + (1|
pop_name)
+(1|Add_yeast:pop_name)+(1|Add_sugar:pop_name)+(1|Add_yeast:Add_sugar:pop_name), data
=KPF_KMF_male)->lm_weight_male
plot(lm_weight_male)
qqnorm(resid(lm_weight_male))
qqline(resid(lm_weight_male))
shapiro.test(resid(lm_weight_male))
anova(lm_weight_male)

emmeans(lm_weight_male,c("Add_yeast","Add_sugar"))->lm_weight_male2
pairs(lm_weight_male2)

emmeans(lm_weight_male,c("Add_yeast"),by=c("Add_sugar"))->lm_weight_male2
pairs(lm_weight_male2)

emmeans(lm_weight_male,c("Add_sugar"),by=c("Add_yeast"))->lm_weight_male2
pairs(lm_weight_male2)
```
